# Genome-Wide Identification and Expression Analysis of Malate Dehydrogenase Gene Family in Sweet Potato and Its Two Diploid Relatives

**DOI:** 10.3390/ijms242316549

**Published:** 2023-11-21

**Authors:** Zhenqin Li, Lei Shi, Xiongjian Lin, Binquan Tang, Meng Xing, Hongbo Zhu

**Affiliations:** College of Coastal Agricultural Sciences, Guangdong Ocean University, Zhanjiang 524088, China; lizhenqiny@126.com (Z.L.); slreus@outlook.com (L.S.); l1843204672@outlook.com (X.L.); tang441823@ouutlook.com (B.T.); xm18729371511@163.com (M.X.)

**Keywords:** sweet potato, *Ipomoea trifida*, *Ipomoea triloba*, malate dehydrogenase, abiotic stress, expression analysis

## Abstract

Malate dehydrogenase (MDH; EC 1.1.1.37) plays a vital role in plant growth and development as well as abiotic stress responses, and it is widely present in plants. However, the MDH family genes have not been explored in sweet potato. In this study, nine, ten, and ten *MDH* genes in sweet potato (*Ipomoea batatas*) and its two diploid wild relatives, *Ipomoea trifida* and *Ipomoea triloba*, respectively, were identified. These *MDH* genes were unevenly distributed on seven different chromosomes among the three species. The gene duplications and nucleotide substitution analysis (Ka/Ks) revealed that the *MDH* genes went through segmental duplications during their evolution under purifying selection. A phylogenetic and conserved structure divided these *MDH* genes into five subgroups. An expression analysis indicated that the *MDH* genes were omni-presently expressed in distinct tissues and responded to various abiotic stresses. A transcription factor prediction analysis proved that Dof, MADS-box, and MYB were the main transcription factors of sweet potato *MDH* genes. These findings provide molecular features of the MDH family in sweet potato and its two diploid wild relatives, which further supports functional characterizations.

## 1. Introduction

As it is an oxidoreductase ubiquitous in plants, animals, and microorganisms, malate dehydrogenase (MDH) catalyzes the reversible conversion of malate to oxaloacetate (OAA) with nicotinamide adenine dinucleotide (NAD+) or nicotinamide adenine dinucleotide phosphate (NADP+) as a coenzyme factor [1]. NAD-dependent MDH is distributed in the cytoplasm, chloroplasts, plastids, mitochondria, peroxisomes, and other microsomes, while NADP-dependent MDH is distributed in chloroplasts [2,3]. Based on metabolic pathways and subcellular localizations, MDH is mainly divided into five subtypes: cytoplasmic MDH (cyMDH) plays a key role in the tricarboxylic acid cycle [4,5]; mitochondrial MDH (mMDH) is involved in acid metabolism within plant tissues and carbon dioxide fixation in C4 plants [6]; peroxisome MDH (pMDH) is involved in photorespiration and in fatty acid β-oxidation [7]; plastidial NADP-dependent MDH (pdNADP-MDH) is considered a key enzyme in the malate valve, which outputs excess reducing equivalents in chloroplasts as malate [1,8,9]; and plastidial NAD-dependent MDH (pdNAD-MDH) mainly plays a role in chloroplasts and non-green plastids in the dark [10]. Polymerase MDHs generally exist as stable dimers or tetramers of the same subunit composition [11].

So far, MDHs have been identified in various plants, including 9 MDHs in *Arabidopsis* [10,12], 12 in rice [13], 13 in cotton [14], 16 in soybean [15], 12 in tomato [16], 8 in common bean [17], 12 in apple [18], 16 in poplar [19], and 12 in Chinese fir [20]. In *Arabidopsis*, *NAD-MDHs* and *cMDHs* have been found to play important roles in embryonic development [12], seed development [21,22], leaf respiration [3], photo respiration [23], energy metabolism [24], and the response to oxidative stress [25,26,27]. Several reports indicate that *MDHs* are involved in abiotic stress responses. Under a salinity treatment, the overexpression of *SbNADP-ME* increases the *Arabidopsis* germination rate and root length, which improves the seedling salt tolerance [28]. The overexpression of *ZmNADP-MDH* improves photosynthesis and salt tolerance in *Arabidopsis* [29]. In apples, *MdcyMDH* overexpression enhances the tolerance to cold and salt stresses by producing more reductive redox states and increasing the salicylic acid level [30,31]. Overexpressing *OsMDH12* rice is susceptible to salt stress, but the *OsMDH12* mutant exhibits enhanced salt endurance [32]. *MDH* genes responding to salt stress are also found in tomato [16] and poplar [19]. In addition, *MDH* genes participate in regulating the nutrient deficiency response. Chinese fir 8 *ClMDH* genes are upregulated under low-phosphorus stress [20]. The cotton *GhmMDH1*-overexpressing plants produce significantly longer roots and have higher biomasses and phosphorus contents than WT plants under phosphorus deficiency [33]. *MDH* genes are involved in aluminum stress response; for example, the overexpression of malate dehydrogenase in alfalfa [34] and tobacco [35] confers tolerance to aluminum.

Being the seventh largest food crop in the world, sweet potato (*I. batatas* (L.) Lam) is also an important energy crop for its high edible, feed, and medicinal values [36]. Sweet potato is hexaploid (2n = 6x = 90) [37], with complex genomes and hybrid incompatibility. The yield and quality of sweet potato are also seriously threatened by abiotic stresses such as salinity and drought, thus resulting in greater economic losses [38]. Molecular breeding is used to cultivate highly resistant sweet potato varieties, which is of great significance for improving the sweet potato yield. At present, the genome-wide identification of *MDH* gene families has been carried out in various plants, but there has not been a report about sweet potato *MDH* genes. In recent years, highly assembled genomes of sweet potato hexaploid [37] and two diploid wild relatives, *I. trifida* (2n = 2x = 30) and *I. triloba* (2n = 2x = 30) [39], have been released. Thus, this work aimed to perform an extensive bioinformatics and expression profiling of the *MDH* gene of sweet potato and its two diploid relatives to reveal their additional biological functions.

In this study, 29 *MDH* genes from sweet potato and its two diploid wild relatives were identified with high confidence. A collinear analysis showed that segmental duplication was the main driver of the evolution of sweet potatoes and their relatives. An evolutionary analysis was performed for these *MDH* genes. Moreover, an expression profiling analysis revealed that these *MDH* genes were expressed in distinct tissues and actively responded to distinct environmental stresses, such as salinity, drought, and low temperatures. Overall, our findings provide a theoretical basis to further investigate the functions of sweet potato *MDH* genes.

## 2. Results

### 2.1. Identification of MDHs in Sweet Potato and Its Two Diploid Wild Relatives

A total of nine, ten, and ten *MDHs* were identified in *I. batatas*, *I. trifida*, and *I. triloba*, respectively, and they were named *IbMDH 1-9, ItfMDH 1-10*, and *ItbMDH 1-10* according to their locations on the chromosomes. In sweet potato, the CDS length of *IbMDHs* ranged from 981 bp (*IbMDH2*) to 1326 bp (*IbMDH9*), and the genomic length varied from 2040 bp to 4842 bp. The lengths of the protein sequences ranged from 326 aa (IbMDH2) to 441 aa (IbMDH9), with a molecular weight (MW) of 34.59 kDa to 48.18 kDa. The isoelectric points (pI) ranged from 6.10 (IbMDH2) to 8.74 (IbMDH7). Most IbMDHs were stable with an instability index of less than 40. The GRAVY values of IbMDHs varied from −0.182 to 0.211. A subcellular localization prediction showed that most IbMDHs were in the chloroplast, IbMDH1, and IbMAH3 in the cytoplasm, and IbMDH7 in the mitochondria (Table 1).

In *I. trifida*, the CDS length ranged from 999 bp to 1329 bp, and the genomic length varied from 2234 bp to 5058 bp. The length of the protein sequences ranged from 332 aa to 442 aa, with an MW of 35.22 kDa to 48.25 kDa and a pI of 6.11 to 8.89. Seven ItfMDHs were stable proteins with an instability index of less than 40. The GRAVY values varied from −0.172 to 0.177, where only ItfNDH5 was a hydrophilic protein. Most of the ItfMDHs were in the chloroplast (Table 1). In *I. triloba*, the CDS length ranged from 999 bp to 1329 bp, and the genomic length varied from 2307 bp to 5829 bp. The length of the protein sequences ranged from 332 aa to 442 aa, with an MW of 35.21 kDa to 48.28 kDa and a pI of 6.11 to 8.79. Eight ItbMDHs were stable proteins. The GRAVY values varied from −0.172 to 0.177. The ItbMDHs were in the chloroplast, mitochondrion, and cytoplasm, respectively (Table 1).

**Figure 1:**
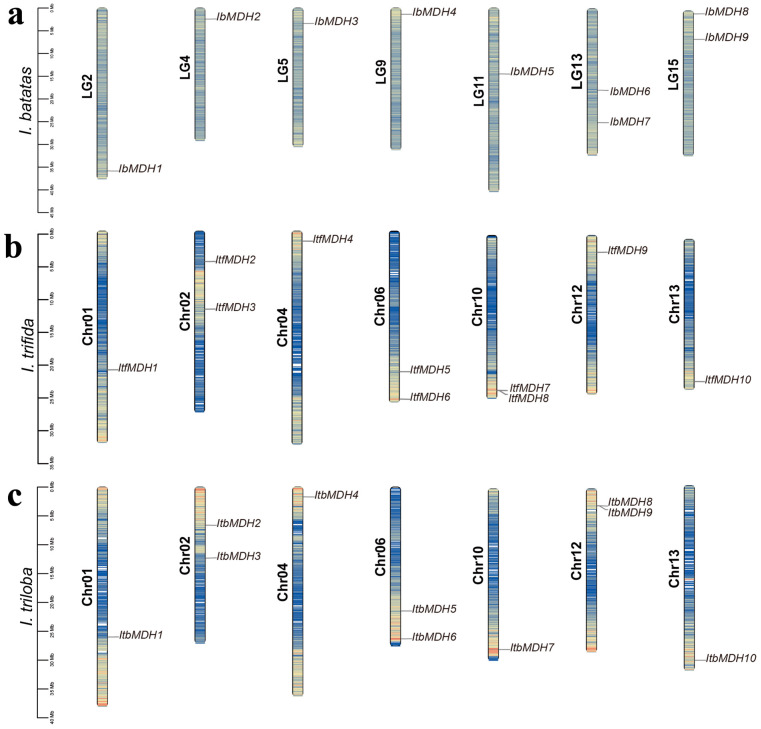
Chromosomal localization and distribution of *MDHs* in *I. batatas* (**a**), *I. trifida* (**b**), and *I. triloba* (**c**). The bars on the left margin represent chromosomes. The chromosome numbers are displayed on the left side of the chromosomes, and the gene names are displayed on the right side. The blue, yellow, and red represent the degree of gene density on the chromosome, from small to large, respectively. Detail chromosomal location information is listed in Table S1.

The chromosomal localizations showed that the *MDHs* from *I. batatas*, *I. triloba*, and *I. trifida* were unevenly distributed across seven chromosomes, respectively (Figure 1 and Table S1). Some chromosomes contained two *MDH* genes, such as LG13 and LG15 in *I. batatas*; Chr02, Chr06, and Chr10 in *I. trifida*; and Chr02, Chr06, and Chr12 in *I. triloba* (Figure 1).

### 2.2. Collinearity Analysis of MDH Genes and Ka/Ks Analysis

**Figure 2:**
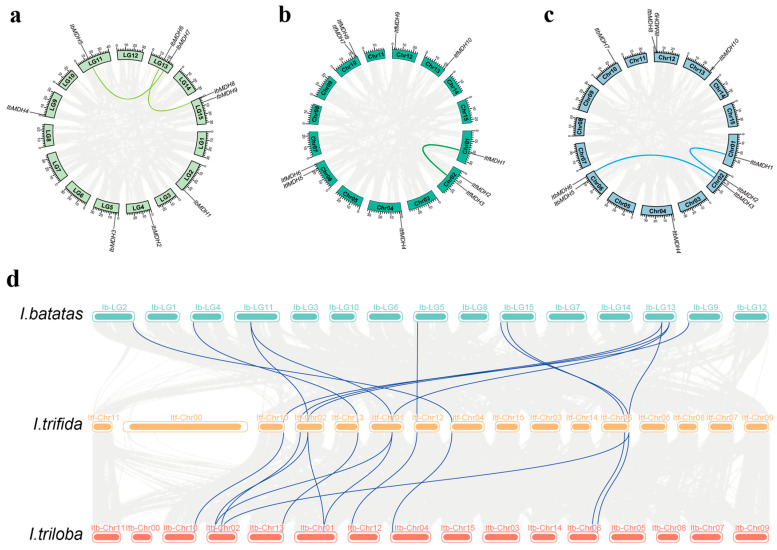
Gene location and collinearity analysis of the *MDH* genes in *I. batatas* (**a**), *I. trifida* (**b**), and *I. triloba* (**c**). The genes were located on different chromosomes. Duplicated gene pairs are linked with a colored line. (**d**) Syntenic analysis of *I. batatas*, *I. trifida*, and *I. triloba MDHs*. Mint green, orange, and red blocks denote chromosomes of *I. batatas*, *I. trifida*, and *I. triloba*, respectively. Foggy blue curves represent the syntenic relationships among the three species.

Gene duplications including tandem or segmental duplications greatly contribute to the diversity and evolutionary history of gene families and play crucial roles in understanding the adaptive evolution of species. It was found that *I. batatas*, *I. trifida*, and *I. triloba* generated two, one, and two pairs of segmental duplication events distributed on the corresponding chromosomes, respectively, i.e., *IbMDH5* and *IbMDH7*, *IbMDH6* and *IbMDH8*, *ItfMDH1* and *ItfMDH3*, *ItbMDH1* and *ItbMDH2*, and *ItbMDH3* and *ItbMDH6* (Figure 2). Additionally, the synteny among three species was investigated. Most *IbMDHs* possessed one orthologous gene with *ItfMDHs* and *ItbMDHs*, except for *IbMDH5*, *IbMDH6*, and *IbMDH7*, which had two (Figure 2d). These orthologous gene pairs were located on different chromosomes, which showed that the duplication in the *MDH* gene family contributed to the process of evolution from diploid to hexaploid.

Furthermore, the Ka/Ks ratios were calculated to estimate the selection pressure and divergence times between the duplicated *MDH* genes (Table 2 and Table S2). The Ka/Ks ratios showed that the duplicated *MDH* genes varied from 0 to 0.71, with an average of 0.12, which implied that these *MDHs* suffered from the influence of purifying selection over the evolution process (Table 2 and Table S2). The Ks values were used to predict the differentiation time of the *MDH* genes’ duplication events. The doubling time of the *MDHs* within each species occurred between 45.4 and 57.2 million years ago (Mya) (Table 2), and the divergence time between sweet potato and *I. trifida* occurred between 0.8 and 57.6 Mya, the divergence time between sweet potato and *I. triloba* occurred between 1.4 and 57.2 Mya, and the divergence time between *I. trifida* and *I. triloba* occurred between 1.5 and 51.1 Mya (Table S2). 

### 2.3. Phylogenetic Relationship Analysis of MDHs in Sweet Potato and Its Two Diploid Wild Relatives

**Figure 3:**
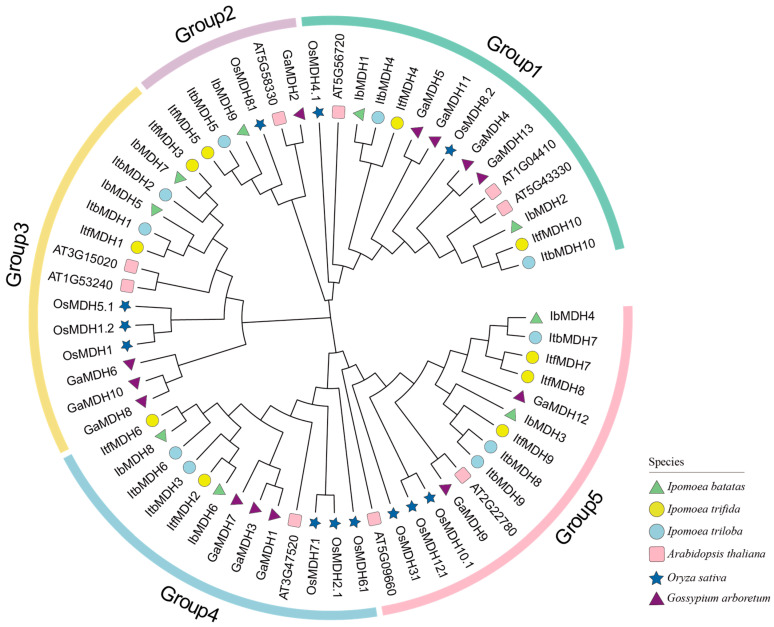
Phylogenetic analysis of *MDHs* in *I. batatas*, *I. triloba*, *I. trifida*, *A. thaliana*, *O. sativa*, and *G. arboretum*. Based on the evolutionary distance, a total of 63 MDHs were divided into five groups (Groups 1, 2, 3, 4, and 5 filled with green, purple, yellow, blue, and pink, respectively). The green triangles represent nine IbMDHs in *I. batatas*. The yellow circles represent ten ItfMDHs in *I. trifida*. The blue circles represent ten ItbMDHs in *I. triloba*. The pink squares represent nine AtMDHs in *A. thaliana*. The dark blue stars represent 12 OsMDHs in *O. sativa*. The purple triangles represent 13 GaMDHs in *G. arboretum*.

To analyze the evolutionary relationships of the *MDH* gene family among *I. batatas*, *I. trifida*, *I. triloba*, *Arabidopsis thaliana*, *Oryza sativa*, and *Gossypium arboreum*, a phylogenetic tree for 63 MDHs of these six species (i.e., 9 in *I. batatas*, 10 in *I. trifida*, 10 in *I. triloba*, 9 in *A. thaliana*, 12 in *O. sativa*, and 13 in *G. arboreum*) was constructed. The results showed that all of the MDHs were divided into five groups (Figure 3). The specific distributions of the MDHs were as follows (total: *I. batatas*, *I. trifida*, *I. triloba*, *A.thaliana*, *O. sativa*, and *G. arboreum*): Group 1 (15: 2, 2, 2, 3, 2, 4), Group 2 (6: 1, 1, 1, 1, 1, 1), Group 3 (14: 2, 2, 2, 2, 3, 3), Group 4 (13: 2, 2, 2, 1, 3, 3), and Group 5 (15: 2, 3, 3, 2, 3, 2) (Figure 3). These results indicated that all sweet potato *MDH* genes were clustered with their corresponding orthologs in *I. triloba* and *I. trifida*.

### 2.4. Conserved Motif and Exon-Intron Structure Analysis of MDHs in Sweet Potato and Its Two Diploid Wild Relatives

**Figure 4:**
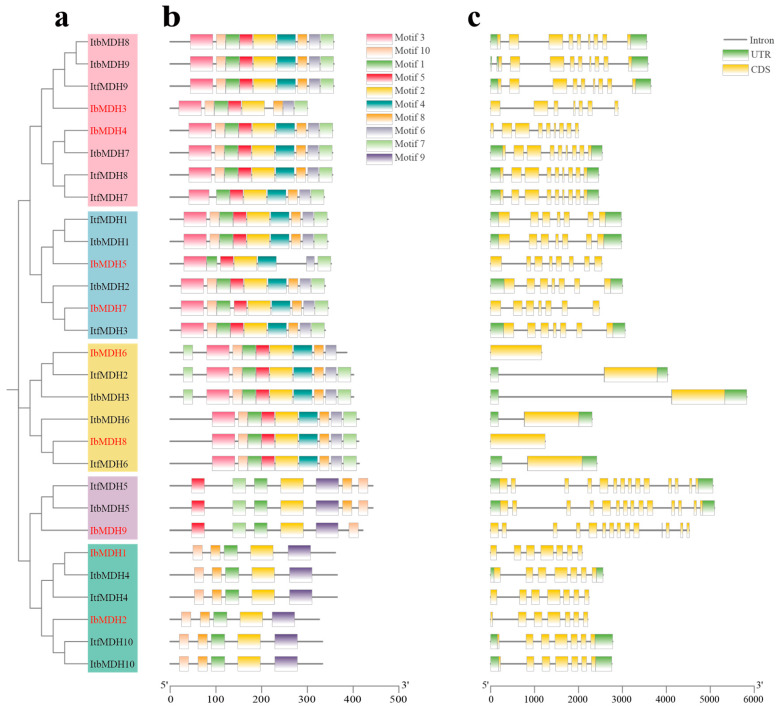
Conserved motifs and exon–intron structure analysis of *MDHs* in *I. batatas*, *I. trifida*, and *I. triloba*. (**a**) The phylogenetic tree of IbMDHs, ItfMDHs, and ItbMDHs. (**b**) The ten conserved motifs were shown in different colors. (**c**) Exon–intron structures of *MDHs*. The green boxes, yellow boxes, and black lines represent UTRs, exons, and introns, respectively.

The distribution of the conserved motifs prediction using the MEME website found that ten conserved motifs were identified containing 15 to 50 amino acids (Figure 4a and Figure S1). Most of the *MDHs* contained these ten conserved motifs but varied within different subgroups. The *MDH* genes in Group 1 missed five motifs (i.e., motifs 3, -4, -5, -6, and -7); in Group 2, the MDHs missed motif 3, motif 4, and motif 6, where *IbMDH9* in Group 2 also missed motif 8; in Group 3, the MDHs missed motif 9; in Group 4 and Group 5, the MDHs lacked motif 9, where *IbMDH5* in Group 4 missed motif 6 and *IbMDH3* in Group 5 missed motif 4. Therefore, the *MDH* genes showed some differences throughout their evolutions.

According to the exon-intron distributions, the quantities of exons and introns in the *MDHs* showed some variations (Figure 4b). The number of introns in the *MDH* genes ranged from 0 to 13, where *IbMDH7* had no introns. The number of exons in the *MDHs* ranged from 1 to 14. The *MDHs* in Group 1 and Group 4 possessed 7 exons, Group 2 contained 14 exons, Group 3 contained 1 exon, and Group 5 contained 9 exons, apart from *IbMDH3*, which contained 7 (Figure 4b). These results indicated that the differences in the evolution and function between the *MDH* members were related to their motifs and the exon and intron differences.

### 2.5. Putative Cis-Regulatory Element Analysis in the Promoter of MDHs in Sweet Potato and Its Two Diploid Wild Relatives

**Figure 5:**
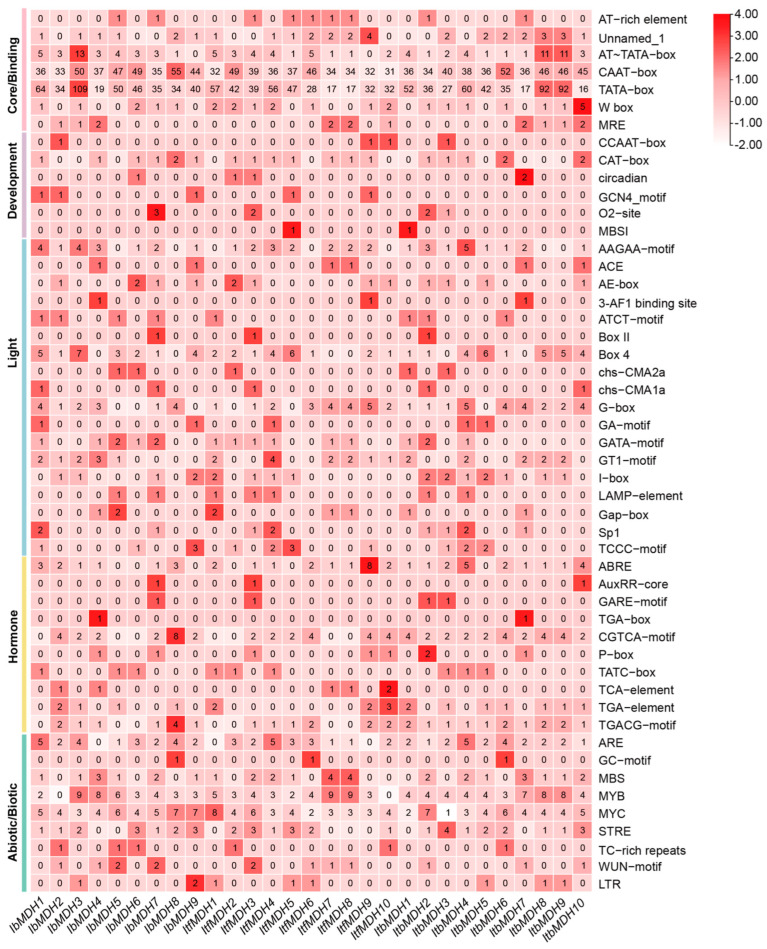
Cis-element analysis in the promoters of *MDHs* from *I. batatas*, *I. trifida*, and *I. triloba*. The cis elements were divided into six broad categories. The degree of red color represents the number of cis elements in the promoters of *MDHs*.

Promoter cis-elements in plants initiate the gene functions related to plant development, hormone regulation, and stress response. Therefore, a cis-element analysis was performed using the 2 kb promoter region of *IbMDHs* (Figure 5 and Table S3). All of the *MDH* genes possessed several hormone-responsive elements, including a CGTCA, TGACG motif, ABRE, TGA-element, and TATC-box (Figure 5). In addition, some abiotic stress-responsive elements, such as the essential anaerobic induction element, ARE; the drought-responsive element and salt-responsive elements, MBS, MYB, and MYC; and the stress response promoter element, STRE, were found to be abundant in the promoters of the *MDH* genes. In addition, the biotic stress-responsive elements were identified in most *MDH* genes, such as WRE3 and the WUN motif (Figure 5 and Table S3). These results suggested that *MDH* genes may be involved in the regulation of hormonal crosstalk and abiotic stress resistance.

### 2.6. Expression Analysis of MDHs in Sweet Potato and Its Two Diploid Wild Relatives

#### 2.6.1. Expression Analysis in Various Tissues

**Figure 6:**
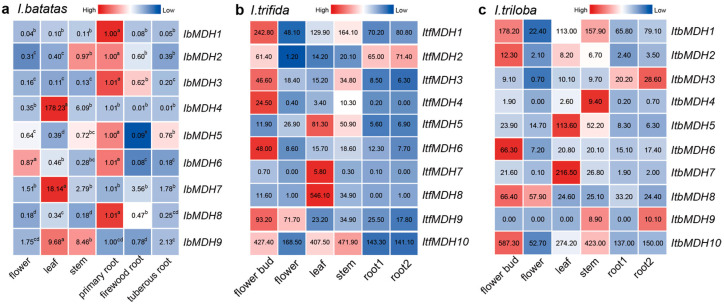
Expression analysis of *MDHs* in different tissues of *I. batatas*, *I. trifida*, and *I. triloba* using RNA-seq. (**a**) Expression analysis of *IbMDHs* in different tissues. The values were determined via qRT-PCR from three biological replicates consisting of pools of three plants, and the results were analyzed using the comparative CT method. The expression level of each *IbMDH* gene in primary root is set as control. Different lowercase letters indicate a significant difference in each *IbMDH* at *p* < 0.05 based on one-way ANOVA followed by Tukey’s post hoc test. (**b**) Expression patterns of *ItfMDHs* in the flower bud, flower, leaf, stem, root 1, and root 2 of *I. trifida*. (**c**) Expression patterns of *ItbMDHs* in the flower bud, flower, leaf, stem, root 1, and root 2 of *I. triloba*. The fragments per kilobase per million (FPKM) values are shown in the color blocks.

The transcript levels of these *IbMDHs* in sweet potato, the flowers, the leaves, stems, primary roots, firewood roots, and tuberous roots were examined by using qPCR (Figure 6a). The results revealed that six *IbMDHs* (i.e., *IbMDH1*, *-2*, *-3*, *-5*, *-6*, and *-8*) were higher in the primary roots than in other tissues. Additionally, the tissue-specific expression pattern was observed. For instance, the *IbMDH4* and *IbMDH8* genes were expressed only in the leaves, and the *IbMDH9* transcript was abundant in the leaves and stems. 

The expression profiles of *MDHs* in six tissues (i.e., flower bud, flower, stem, leaf, root 1, and root 2) of *I. trifida* and *I. triloba* were analyzed (Figure 6b,c). In *I. trifida*, seven *ItfMDHs* genes were highly expressed in the flower buds, such as *ItfMDH1* and *ItfMDH6*. Meanwhile, *ItfMDH7* and *ItfMDH8* appeared to be tissue-specific and were expressed only in the leaves. In addition, the *ItfMDH10* transcript accumulated in the flower buds and leaves as well as the stems (Figure 6b). Similarly, in *I. triloba*, the five *ItbMDHs* were more highly expressed in the flower buds; *ItbMDH3* and *ItbMDH9* were highly expressed in root 2; and the transcripts of *ItbMDH5* and *ItbMDH7* accumulated mainly in the leaves. Furthermore, one gene, *ItbMDH4*, was specifically expressed only in the stems (Figure 6c).

#### 2.6.2. Expression Analysis under Potassium Deficiency in Sweet Potato

**Figure 7:**
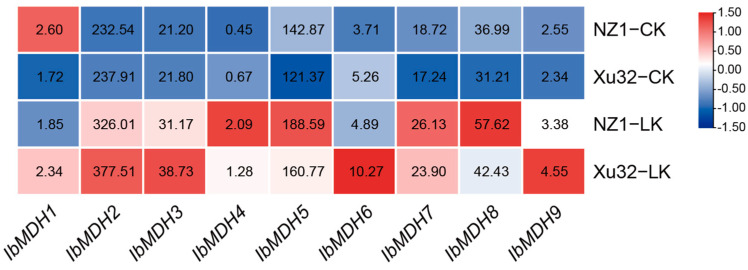
Expression analysis of sweet potato *MDH* genes under potassium deficiency as determined via RNA-seq. NZ1: “Ningzishu 1”; Xu32: “Xushu 32”. The FPKM values are shown in the color blocks.

As shown in Figure 7, the expression profiles of the nine sweet potato *MDH* genes were detected using the transcriptome data of low-k-tolerant “Xushu 32” and low-k-sensitive “Ningzishu 1” under potassium deficiency from the NCBI database (PRJNA1013090)**.** Under a low-potassium treatment, except for *IbMDH1*, which was upregulated in “Xushu 32” and downregulated in “Ningzishu 1”, the transcript levels of eight sweet potato *MDH* genes were significantly upregulated in both varieties (Figure 7). 

#### 2.6.3. Expression Analysis under Hormone Stress

**Figure 8:**
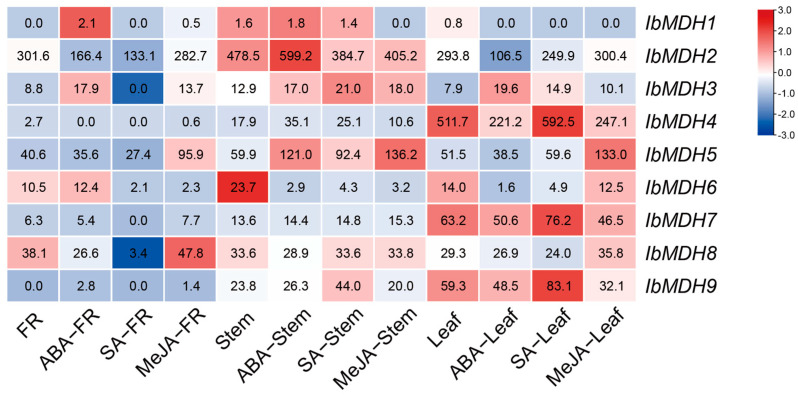
Expression analysis of sweet potato *MDH* genes in fibrous roots (FR), stems, and leaves of sweet potato under hormone treatments as determined via RNA-seq. The FPKM values are shown in the color blocks.

The expression profiles of nine sweet potato *MDH* genes were identified in three distinct tissues under ABA, SA, and MeJA treatments using the “Xushu 18” RNA-seq data obtained from the NCBI database (PRJNA511028) (Figure 8). In fibrous roots, *IbMDH1* and *IbMDH3* were upregulated after the ABA treatment, and *IbMDH5* and *IbMDH8* were upregulated after the MeJA treatment, whereas the others were downregulated. The *IbMDHs* in stems exhibited varied differential expressions during the hormone treatments. For example, the transcript of *IbMDH6* was downregulated under all three hormone treatments, *IbMDH2* was upregulated only under the ABA treatment, and *IbMDH1* was downregulated only under the MeJA treatment. In the leaves, except for *IbMDH3*, all eight *IbMDHs* were downregulated under the ABA treatment; four *IbMDHs* were upregulated and four *IbMDHs* were downregulated upon the SA treatment; and *IbMDH5* was significantly induced under the MeJA treatment, whereas *IbMDH4*, *IbMDH7*, and *IbMDH9* were downregulated (Figure 8).

The expression patterns of *ItfMDHs* and *ItbMDHs* were also analyzed using the RNA-seq data of *I. trifida* and *I. triloba* under the ABA, GA3, and IAA treatments [39] (Figure S2). After the ABA treatment, the transcripts of five *ItfMDHs* (*ItfMDH1*, *-5*, *-6*, *-9*, and *-10*) were strongly induced, and only *ItfMDH8* was downregulated, and the others did not show significant changes. Under the GA3 treatment, *ItfMDH4* and *ItfMDH7* were upregulated. Under the IAA treatment, *ItfMDH3* was upregulated. In *I. triloba*, under the ABA treatment, *ItbMDH4* and *ItbMDH8* were upregulated, and the others were downregulated. Under the GA3 treatment, *ItbMDH3* was upregulated, and *ItbMDH9* was downregulated. Under the IAA treatment, *ItbMDH7* was upregulated, and *ItbMDH1*, *ItbMDH4*, and *ItbMDH10* were downregulated (Figure S2).

#### 2.6.4. Expression Analysis under Cold Stress

**Figure 9:**
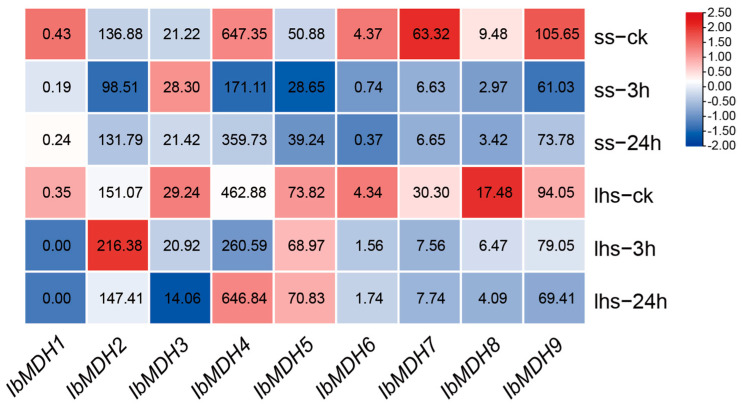
Gene expression patterns of sweet potato *MDH* genes under cold stress as determined via RNA-seq. ss: cold-sensitive “Shenshu 28”; lhs: cold-tolerant “Liaohanshu 21”. The FPKM values are shown in the color blocks.

As shown in Figure 9, the expression profiles of the nine *IbMDHs* were detected using the transcriptome data of cold-tolerant “Liaohanshu 21” and cold-sensitive “Shenshu 28” after cold stress [40]. *IbMDH2* and *IbMDH4* were upregulated in “Liaohanshu 21” under cold stress, whereas *IbMDH3* was upregulated in “Shenshu 28”, and other *IbMDHs* were downregulated or did not exhibit significant changes in both sweet potato cultivars (Figure 9).

Additionally, the expression patterns of *ItfMDHs* and *ItbMDHs* were analyzed under cold stress (Figure S3). Except for *ItfMDH1*, *ItfMDH2*, *ItfMDH4*, and *ItfMDH6*, which were upregulated at low temperatures, the other six *ItfMDHs* genes were downregulated. After a cold treatment, *ItbMDH3*, *ItbMDH4*, *ItbMDH6*, and *ItbMDH8* were induced, whereas the others were downregulated.

#### 2.6.5. Expression Analysis under Heat Stress

**Figure 10:**
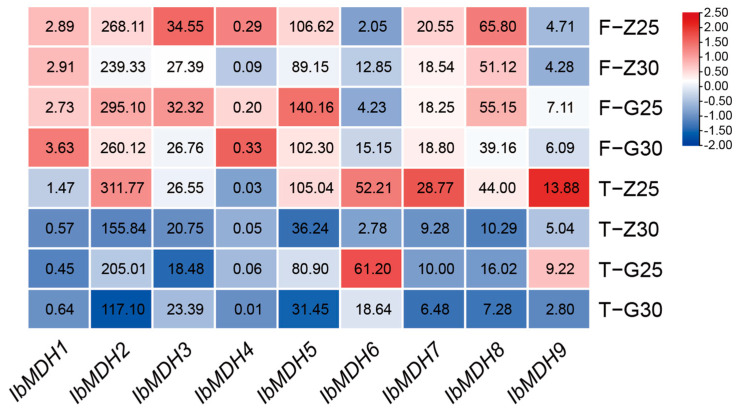
Gene expression patterns of sweet potato *MDH* genes under heat stress as determined via RNA-seq. F: fibrous roots; T: tuberous roots; Z: heat-sensitive “Ziluolan”; G: heat-tolerant “Guangshu 87”. The FPKM values are shown in the color blocks.

The expression profiles of the nine sweet potato *MDH* genes were detected using the transcriptome data of heat-tolerant “Guangshu 87” and heat-sensitive “Ziluolan” after heat stress (Figure 10). After a heat treatment, the *MDH* gene transcripts of fibrous roots and tuberous roots were downregulated in the “Ziluolan” cultivar, but in the “Guangshu 87” cultivar, only *IbMDH1*, *IbMDH4*, and *IbMDH6* in fibrous roots were upregulated, while the other *IbMDHs* were downregulated.

The expression patterns of *ItfMDHs* and *ItbMDHs* were also analyzed under heat stress (Figure S4). After a heat treatment, except for *ItfMDH3* and *ItfMDH6*, eight *ItfMDHs* were upregulated. Six *ItbMDHs* were upregulated in *I. triloba*, while *ItbMDH2*, *ItbMDH4*, *ItbMDH5*, and *ItbMDH9* were downregulated.

#### 2.6.6. Expression Analysis under Salt and Drought Stresses

**Figure 11:**
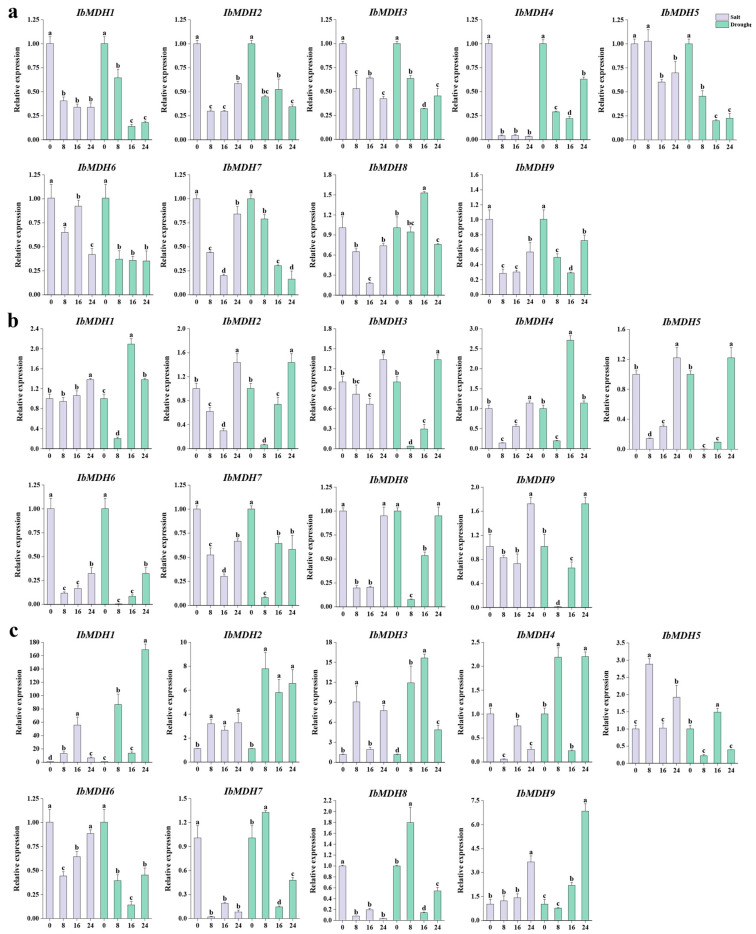
Expression analysis of *IbMDHs* in different tissues under salt and drought treatments. (**a**) Root. (**b**) Stem. (**c**) Leaf. The values were determined via qRT-PCR from three biological replicates consisting of pools of three plants, and the results were analyzed using the comparative CT method. The expression at 0 h in each treatment was determined as control. Different lowercase letters indicate a significant difference in each *IbMDH* at *p* < 0.05 based on one-way ANOVA followed by Tukey’s post hoc test.

The qPCR was performed to analyze the expression levels of nine *IbMDHs* after salt and drought treatments in the primary roots, stems, and leaves for 8 h, 16 h, and 24 h, respectively (Figure 11). The results showed significant changes in the expressions of *IbMDHs* in different tissues after both stresses. In the primary roots, compared to the control, all *IbMDHs* except for *IbMDH8* were upregulated upon drought stress, and the transcript levels of eight *IbMDH* genes were downregulated under both stresses (Figure 11a). In the stems, six *IbMDHs* (i.e., *IbMDH1*, *-2*, *-3*, *-4*, *-5*, and *-9*) were upregulated upon both stresses, showing similar expression patterns (Figure 11b). Additionally, in the leaves, five *IbMDHs* (i.e., *IbMDH1*, *-2*, *-3*, *-5*, and *-9*) were upregulated after both stresses, and *IbMDH4*, *IbMDH7*, and *IbMDH8* were upregulated only under drought stress (Figure 11c). Notably, the expression of *IbMDH1* was increased by 55-fold at 16 h of salt stress and by 168-fold at 24 h of drought stress, and *IbMDH3* was significantly upregulated by 7-fold at 8 h of salt stress and by 12-fold at 16 h under drought stress compared to the control (Figure 11c). These results indicated that *IbMDH1* and *IbMDH3* could be important candidate genes involved in salt and drought stress responses. 

To understand the possible roles of *ItfMDHs* and *ItbMDHs*, the expression patterns of *I. triloba* and *I. trifida* were examined under drought and salt stresses [39]. In *I. trifida*, *ItfMDH5* and *ItfMDH9* were upregulated under both stresses. In *I. triloba*, *ItbMDH5* and *ItbMDH6* were upregulated, and *ItMDH7* was downregulated under both stresses (Figure S5).

### 2.7. Protein Interactions Network of MDHs in Sweet Potato

**Figure 12:**
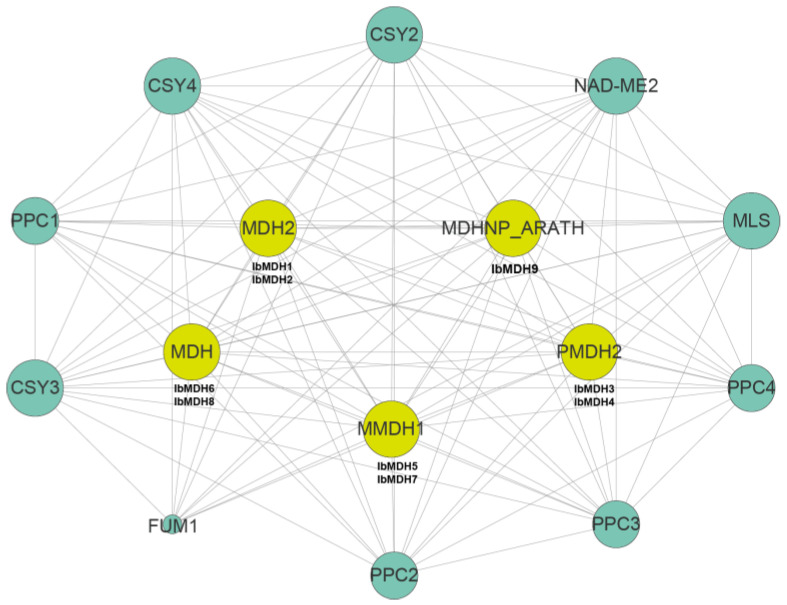
Protein-protein interaction network of sweet potato MDH proteins; the size and color of the circle represent interaction degree.

A sweet potato MDH protein interactions network was constructed based on the *Arabidopsis* protein interactions model (Figure 12 and Table S4). The protein interaction predictions indicated that all nine IbMDHs were orthologous with the *Arabidopsis* proteins. For example, IbMDH1 and IbMDH2 were homologous with MDH2, IbMDH3 and IbMDH4 were homologous with PMDH2, IbMDH5 and IbMDH7 were homologous with mMDH1, IbMDH6 and IbMDH8 were homologous with MDH, and IbMDH9 were homologous with MDHNP_ARATH. Interestingly, these IbMDHs interacted with each other. In addition, these IbMDHs also interacted with other functional proteins, such as Citrate synthases CSY2, CSY3, and CSY4, Phosphoenolpyruvate carboxylases PPC1, PPC2, PPC3, and PPC4, and Malate synthase MLS. These results indicated that *IbMDHs* played key roles in the regulation of the TCA cycle pathway in the sweet potato. 

### 2.8. Transcript Factors Network of Sweet Potato MDH Genes

**Figure 13:**
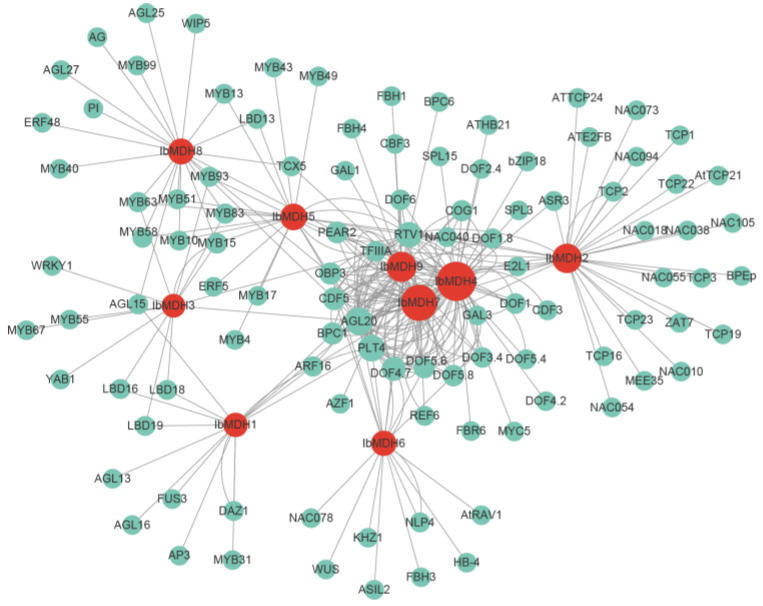
The putative transcription factor regulatory network analysis of sweet potato *MDH* genes. Turquoise circular nodes represent transcription factors; red circular nodes represent *IbMDHs*; and node size represents the degree of interaction between nodes based on degree value.

The potential TF analysis revealed that among the *IbMDHs*, a total of 275 TFs were identified, distributing in 25 different TF families (i.e., AP2, ARF, B3, BBR-BPC, bHLH, bZIP, C2H2, C3H, CPP, Dof, E2F/DP, ERF, LBD, MADS-box, MYB, NAC, Nin-like, RAV, SBP, TCP, Trihelix, WOX, WRKY, YABBY, and ZF-HD) (Figure 13 and Table S5). Among them, Dof, with 84 members, was the most highly enriched, followed by MADS-box (35), MYB (29), AP2 (23), and B3 (21). Among all nine *IbMDHs*, *IbMDH4* was the most targeted by 63 TFs, followed by *IbMDH7* (53), *IbMDH9* (34), and *IbMDH2* (32), while *IbMDH3* was targeted least, by only 14 TFs (Figure 13 and Table S5).

## 3. Discussion

### 3.1. Evolution of MDH Genes in Sweet Potato and Its Two Diploid Wild Relatives

Malate dehydrogenase participates in the TCA cycling pathway and plays an important regulatory role in plant growth and development and stress protection, but it has not been reported in sweet potatoes. Due to the complexity of the genome of hexaploid sweet potatoes, its two diploid relatives, *I. trifida* and *I. triloba*, have often been studied [41,42]. In our study, nine, ten, and ten *MDH* genes were identified in *I. batatas*, *I. trifida*, and *I. triloba*, respectively, which is less than rice [13], cotton [14], and tomato [16]. These *MDH* genes were scattered on seven chromosomes, several of which contained two *MDH* genes, such as LG13 and LG15 in sweet potatoes, and Chr02 and Chr06 in *I. trifida* and *I. triloba* (Figure 1), which supported that they might possess different biological regulator functions. Gene replication is one of the main drivers of gene family expansion and genome evolution [43]. Both tandem and segmental repetitions took place in the *MDH* families [13,20]. However, only three, two, and three segmental duplication gene pairs were identified from *I. batatas*, *I. trifida*, and *I. triloba*, respectively (Figure 4a), with Ka/Ks ratios < 1 (Table 2). These results suggest that the expansion of the *MDH* gene family may have been derived from genome polyploidy events to diversify gene function at a purification pressure. During its long-time evolution, sweet potato had a similar number of collinear *MDHs* with *I. trifida* as with *I. triloba* (Figure 4b and Table S2), which is consistent with the previous study [42]. All *MDH* genes had distinct subcellular localizations (chloroplasts, cytoplasm, and mitochondria), which could lead to functional differences. Based on the phylogenetic tree, the *MDH* gene family was divided into five subgroups (Figure 3), concordant with the previous study [16,19,20]. In this phylogenetic tree, each *IbMDH* always showed one orthologous gene in *I. trifida* and *I. triloba*, respectively (Figure 3), implying that the *MDHs* of sweet potato originated from those of its diploid ancestors [37]. The *MDH* subgroup contained the same types and numbers of motifs, introns, and exons (Figure 4b,c), suggesting that the *MDH* members in each subfamily may have similar functions [44].

### 3.2. Distinct Roles of MDH Genes in Biological Processes

The expression profiling of *MDH* genes in different tissues, developmental stages, and under abiotic stresses provides new data that could help in understanding potential biological functions. In general, 67% (6/9) of the sweet potato *MDH* genes showed high expression patterns in the primary roots, and some *MDH* genes exhibited tissue-specific expressions; for instance, *IbMDH4* and *IbMDH8* were expressed only in the leaves, and *IbMDH9* was expressed in the leaves and stems (Figure 6a). Previous studies found that inhibiting the expression of *SlmMDHs* reduced the activity of malate dehydrogenase in plants, reduced the biomass of the root system, and changed the root growth structure [9], so it was speculated that sweet potato *MDH* genes were mainly involved in the growth and development of primary roots. Interestingly, the transcripts of 70% (7/10) *ItfMDHs* and 50% (5/10) *ItbMDHs* were highly expressed in the flower buds, and other *MDH* genes were also expressed in the roots, stems, and leaves (Figure 6b,c), which showed some differences from the *MDHs* in sweet potato. It might be that the *MDH* gene functions changed during the evolution from diploid to hexaploid. 

The *MDH* genes in some plants also respond to nutrient deficiency. The overexpression of the *mMDH* gene improved the tobacco phosphorus access [45]. Under low-phosphorus stress, the overexpression of cotton *GhmMDH1* significantly increased the content of malic acid in the plant roots, leaves, and root exudates [33]. Twelve *ClMDH* genes were identified in Chinese fir to participate in the low-phosphorus stress response [20]. Additionally, phosphorus deficiency triggered the induction of the *AtPPC1* transcript, which was responsible for the phosphorus metabolic adaptations [46] and predicted to interact with the sweet potato MDH proteins (Figure 11). This suggests that sweet potato *MDH* genes may participate in the phosphorus deficiency response, which is consistent with previous studies [20,33]. In our study, under potassium deficiency, 89% (8/9) of the sweet potato *MDH* genes were significantly upregulated in both low-potassium-sensitive “Ningzishu 1” and low-potassium-resistant “Xushu 32”. Additionally, only *IbMDH1* had a different expression with an upregulation in “Xushu 32” but a downregulation in “Ningzishu 1” (Figure 7). These results suggest that sweet potato *MDH* genes strongly respond to potassium deficiency. 

In apples, the suppression of *MdcyMDH* altered SA signaling, indicating their possible roles in the regulation of improving cold and salt tolerance [30]. However, there is no research on *MDHs* regulating other hormones. In this study, the CGTCA and TGACG motifs (MeJA-responsive element) and ABRE (ABA-responsive element) were abundant in the *MDH* gene promoters (Figure 5). Interestingly, most of the *IbMDHs* were induced under the ABA, MeJA, and SA treatments in fibrous roots, stems, and leaves (Figure 8). Most of the *ItfMDHs* and *ItbMDHs* were also induced by the ABA, GA3, and IAA treatments (Figure S2). Particularly, *IbMDH1* and its homologous *ItfMDH4* and *ItbMDH4*, and *IbMDH3* and its homologous *ItfMDH9* and *ItbMDH8* were upregulated after the ABA treatment (Figure S2). These results suggest that the *MDHs* may engage in the hormone crosstalk in sweet potato and its relatives.

In this study, rich ARE, MBS, MYB, MYC, and STRE were identified in the sweet potato *MDH* promoters (Figure 5), implying that sweet potato *MDH* genes responded to various abiotic stresses [47]. The expression profiles showed that the transcripts of all six *MDH* genes were inhibited under cold stress, and only *IbMDH2* and *IbMDH4* were upregulated in the cold-tolerant “Liaohanshu 21”, and *IbMDH3* was upregulated in the cold-sensitive “Shenshu 28” (Figure 9). Similarly, the transcript levels of the pea *NADP-MDH* gene increased under cryogenic duress [48]. Additionally, in the heat-tolerant “Guangshu 87”, *IbMDH1*, *IbMDH4*, and *IbMDH6* in fibrous roots were upregulated under heat stress, while all of the *MDH* genes in heat-sensitive “Ziluolan” were downregulated (Figure 10), similar with the tomato *MDH* genes [16]. The rice plastidial *NAD*-*MDH1* negatively regulated salt treatment by reducing the vitamin B6 content [49]. The functional characterizations indicated that the *MDH* genes were linked to plant salt tolerance [28,29,32]. The sweet potato *MDH* genes in the stems and leaves may play important roles in regulating salt and drought stresses, as shown in Figure 10. Only one transcript of the *IbMDH8* gene in the primary roots was induced by drought stress, whereas 67% (6/9) of the *IbMDHs* in the stems and 56% (5/9) in the leaves were upregulated under both stresses. Notably, sweet potato *MDH* transcripts responded more to drought stress than salt stress. For example, the transcript levels of *IbMDH1* and *IbMDH3* were increased by 168-fold and 12-fold upon drought stress, but increased by 55-fold and 7-fold under salt stress, and *IbMDH4*, *IbMDH7*, and *IbMDH8* in the leaves were upregulated only after drought stress (Figure 11). Interestingly, the protein interaction prediction analysis revealed that the sweet potato MDH proteins interacted with the PEPC proteins (PPC1, PPC2, PPC3, and PPC4) (Figure 12), which were inducted under salt and drought stresses. Particularly, *AtPPC4* showed the highest induction in response to both stresses [50], and the *AtPPC3* mutant showed a stressed phenotype in control circumstances but was unaffected by high salinity [51]. These results indicate that sweet potato *MDH* genes are responses to distinct abiotic stresses.

The *MDH* genes in *I. trifida* and *I. triloba* actively responded to abiotic stress (Figures S2–S5). For example, *ItfMDH5* and *ItbMDH5*, the closest homologs of *IbMDH9*, were strongly upregulated by salinity and drought stress. Similar expression patterns were observed in the *IbMDH8* homologs *ItfMDH6* and *ItbMDH6* and in the *IbMDH6* homologs *ItfMDH2* and *ItbMDH3* in response to cold stress, and similar expression patterns were observed in the *IbMDH4* homologs *ItfMDH7* and *ItbMDH7* and in the *IbMDH2* homologs *ItfMDH10* and *ItbMDH10* in response to heat stress. 

Epigenetic mechanisms play significant roles in the responses of plants to abiotic stresses [52,53]. The *MDH* genes in plants are regulated by epigenetic mechanisms such as DNA methylation. For instance, the high methyl status of the promoters of the light regulation *MDH* genes suppressed their transcription [54]. The regulation of *Zea mays* malate dehydrogenase under hypoxic conditions took place at the epigenetic level by changing the methyl status of the promoters of their genes [55]. This suggests that the *MDH* genes of sweet potato and its relatives may also be regulated by epigenetic mechanisms in response to abiotic stresses. 

## 4. Materials and Methods

### 4.1. Identification and Physicochemical Properties of MDH Family Members

The protein sequences and GFF gene annotation files of sweet potato, *I. trifida*, and *I. triloba* were obtained from the Sweet Potato Genomics Resource (http://sweetpotato.plantbiology.msu.edu/, accessed on 17 July 2023) and Ipomoea Genome Hub (https://ipomoea-genome.org/, accessed on 17 July 2023). The *Arabidopsis* MDH proteins were downloaded from the TAIR database (https://www.arabidopsis.org/, accessed on 17 July 2023), and *Oryza sativa* and *Gossypium arboreum* MDH proteins were from Phytozome v13 (https://phytozome-next.jgi.doe.gov/, accessed on 17 July 2023) [56]. To identify the *MDH* genes, two methods were utilized. First, HMMsearch (ver. 3.1b2) with default settings was used to search the protein sequences of the three species for the MDH domain (Pfam accession numbers: PF00056 and PF02866) [57]. Second, *Arabidopsis* MDH protein sequences were used as queries in a BLASTP (ver. 2.10.0+) search against the protein database of each species with an E-value threshold of 1 × 10^–10^ [58]. Then, all the MDH proteins were subjected to search against the NCBI-CDD search tool (https://www.ncbi.nlm.nih.gov/Structure/bwrpsb/bwrpsb.cgi, accessed on 19 July 2023) [59] and the Simple Modular Architecture Research Tool (SMART) (http://smart.embl-heidelberg.de/, accessed on 19 July 2023) [60]. Finally, with the full-length transcriptome of third-generation sequenced “Guangshu 87” as a reference, the candidate sweet potato MDH family members were manually corrected using SnapGene software 6.0.2. ExPASy ProtParam instrument (http://web.expasy.org/protparam/, accessed on 20 July 2023) was used to calculate protein physicochemical parameters [61]. WoLF PSORT (https://wolfpsort.hgc.jp/, accessed on 20 July 2023) was used to create subcellular localization predictions [62].

### 4.2. Chromosome Distribution and Collinearity Analysis of MDH Genes

TBtools software (v.2.001) [63] was used to visualize the distribution of *IbMDHs*, *ItfMDHs*, and *ItbMDHs* on the chromosomes. The collinearity relationships of *MDH* genes within species were constructed using *MCScanX* [64] and visualized using TBtools software 1.098.

### 4.3. Synteny Analysis and Calculation of Ka/Ks Values of MDH Gene Homologous Pair

The synteny analysis of *MDHs* among the whole genomes of *I. batatas*, *I. trifida*, and *I. triloba* was performed using *MCScanX* [64] and visualized using TBtools software. The nucleotide substitution rates (Ka and Ks) and Ka/Ks ratios of duplicated *MDH* genes were estimated using the TBtools software’s Ka/Ks calculation package. The estimated time of detergence (T, Mya: million years ago) was calculated as described previously [65].

### 4.4. Phylogenetic Analysis of MDH Genes

The phylogenetic analysis of MDHs from *I. batatas*, *I. trifida*, *I. triloba*, *Arabidopsis*, *O. sativa*, and *G. arboreum* was performed using ClustalW in MEGA 7 [66] with default parameters, the neighbor-joining (NJ) method, and the Poisson correction model, and bootstrapping was performed with 1000 replicates. Finally, the online tool Evolview (http://www.evolgenius.info/evolview/, accessed on 28 August 2023) [67] was used to beautify the phylogenetic tree. 

### 4.5. Conserved Motifs and Gene Structure Analysis of MDH Genes

The MEME tool (https://meme-suite.org/meme/, accessed on 28 August 2023) analyzed conserved motifs of MDH proteins, and the maximum number of motifs parameter was set to 10 [68]. The MDHs phylogenetic tree, gene structure, and conserved motifs were shown using the TBtools software 1.098 [63].

### 4.6. Cis-Regulatory Element Analysis of MDH Genes

The upstream promoter region (2000 bp) of the *MDH* genes was extracted using TBtools software and submitted to PlantCARE (https://bioinformatics.psb.ugent.be/webtools/plantcare/html/, accessed on 12 September 2023) website [69], which identified the cis-regulatory elements in the *MDH* genes. Then, the TBtools software was used to visualize the cis-regulatory element figure. 

### 4.7. Protein–Protein Interactions Analysis of Sweet Potato MDH Proteins

Using the default parameters, the online STRING database (https://string-db.org/, accessed on 12 September 2023) was utilized to predict and execute potential protein–protein interaction networks using sweet potato MDH proteins based on known *Arabidopsis* homologs. 

### 4.8. Transcription Factors Regulatory Network Analysis of Sweet Potato MDH Genes

The online tool Plant Transcriptional Regulatory Map (PTRM)^23^ [70] was used to predict TFs in the upstream (1000 bp) regions of sweet potato *MDH* genes with *p* ≤ 1 × 10^–5^. The potential TF regulatory networks were visualized by the Cytoscape software version 3.8 [71].

### 4.9. Transcriptome Analysis

Four transcriptome bio project datasets were chosen for the sweet potato *MDH* gene expression profile analysis. Three bio project datasets (PRJNA1013090 for low potassium, PRJNA511028 for hormone, and PRJNA987163 for cold) were downloaded from the NCBI database. Another one was our in-house (unpublished) sweet potato heat treatment. Among them, “Xushu 18” was used for hormonal treatment, cold-tolerant “Liaohanshu 21” and cold-sensitive “Shenshu 28” were used for cold treatment, low-k-tolerant “Xushu 32” and low-k-sensitive “Ningzishu 1” were usedfor low-potassium treatment, and heat-tolerant “Guangshu 87” and heat-sensitive “Ziluolan” were used for heat treatment. Additionally, the gene expression data in *I. trifida* and *I. triloba* were downloaded from the Sweet Potato Genomics Resource (http://sweetpotato.plantbiology.msu.edu/, accessed on 17 July 2023). The *MDH* expression was measured in fragments per kilobase of exon per million fragments mapped (FPKM) [72]. The heat maps of expression were constructed using TBtools software 1.098.

### 4.10. qRT-PCR Analysis of Sweet Potato MDH Genes

The sweet potato (*I. batatas*) cultivar “Jishu 26” was used for qRT-PCR analysis in this study. Sweet potato plants were cultivated in a field at the experimental field of Guangdong Ocean University, Guangdong, China. For tissue expression, the flower, leaf, stem, primary root, firewood root, and tuberous root tissues were sampled from 3-month-old “Jishu 26” planted in the field. For the abiotic stress treatments, twigs about 30 cm in length from 3-month-old field-grown “Jishu 26” were cultured in the Hoagland solution for 14 days to treat them: for salt stress treatment, the twigs were cultured in the Hoagland solution with 0 and 200 mM NaCl, and for drought stress treatments, the twigs were cultured in Hoagland solution with 0 and 300 mM mannitol. The primary root, stem, and leaf samples were collected at 0, 8, 16, and 24 h after the treatments. 

Total RNA was extracted using the TRIzol method (Invitrogen, Carlsbad, CA, USA). The qRT-PCR experiments were carried out based on previous studies [73]. Three biological replicates were established for each sample. Primers used in the study are listed in Supplemental Table S6, where the *IbARF* gene is an internal reference [74].

## 5. Conclusions

In this study, 29 *MDH* genes were identified in sweet potato and its two diploid relatives. A conserved motif and gene structure analysis showed the conservation and dispersion of *MDHs* during evolution. The expression profiles indicated that the *MDHs* showed tissue specificity and various expression patterns in sweet potato and its two diploid relatives. *MDH* genes may be involved in various hormone and abiotic stress responses to regulate plant growth and development. The findings in this study will help us better understand the biological functions of *MDHs* in coping with climate change and identify possible candidate genes for enhancing field and abiotic stress tolerance in sweet potato and its two diploid relatives.

## Figures and Tables

**Table 1:** Characteristics of *MDHs* in *I. batatas*, *I. trifida*, and *I. triloba*.

Gene Name	Accession Number	Genomic Length (bp)	CDS Length (bp)	Protein Size (aa)	MW (kDa)	Isoelectric Point (pI)	Instability Index	GRAVY	Subcellular Localization
*IbMDH1*	OR359888	3360	1086	361	39.72	6.27	42.77	−0.008	Cytoplasm
*IbMDH2*	OR359889	2474	981	326	34.59	6.1	34.3	0.077	Chloroplast
*IbMDH3*	OR359890	3567	999	332	34.69	7.54	32.43	0.211	Cytoplasm
*IbMDH4*	OR359891	2227	1068	355	37.27	7.57	30	0.196	Chloroplast
*IbMDH5*	OR359893	2749	1035	344	35.98	8.58	35.63	0.126	Chloroplast
*IbMDH6*	OR359895	4394	1161	386	41.12	8.45	41.48	−0.006	Chloroplast
*IbMDH7*	OR359896	2726	1041	346	35.98	8.74	34.19	0.133	Mitochondrion
*IbMDH8*	OR359898	2040	1239	412	43.69	6.55	41.85	0.034	Chloroplast
*IbMDH9*	OR359899	4842	1326	441	48.18	7.07	27.27	−0.182	Chloroplast
*ItfMDH1*	OR359902	2973	1035	344	35.99	8.79	36.16	0.122	Mitochondrion
*ItfMDH2*	OR359900	4025	1203	400	42.57	7.57	40.29	0.017	Chloroplast
*ItfMDH3*	OR359911	3058	1017	338	35.22	8.89	36.09	0.117	Mitochondrion
*ItfMDH4*	OR359906	2234	1095	364	39.92	6.47	42.56	0.015	Chloroplast
*ItfMDH5*	OR359908	5058	1329	442	48.25	6.73	28.78	−0.172	Chloroplast
*ItfMDH6*	OR359912	2416	1239	412	43.68	6.55	41.97	0.052	Chloroplast
*ItfMDH7*	OR359913	2457	1011	336	35.52	8.76	30.44	0.176	Mitochondrion
*ItfMDH8*	OR359901	2457	1065	354	37.27	8.4	29.26	0.177	Mitochondrion
*ItfMDH9*	OR359905	3647	1074	357	37.51	8.56	34.95	0.127	Chloroplast
*ItfMDH10*	OR359904	2777	999	332	35.53	6.11	32.35	0.045	Cytoplasm
*ItbMDH1*	OR359916	2978	1035	344	35.99	8.79	36.16	0.122	Mitochondrion
*ItbMDH2*	OR359926	2999	1017	338	35.21	8.54	38.46	0.133	Mitochondrion
*ItbMDH3*	OR359919	5829	1203	400	42.58	8.43	38.83	0	Chloroplast
*ItbMDH4*	OR359925	2558	1095	364	39.93	6.27	42.24	0.013	Cytoplasm
*ItbMDH5*	OR359923	5094	1329	442	48.28	6.48	28.82	−0.172	Chloroplast
*ItbMDH6*	OR359915	2307	1239	412	43.71	6.96	42.71	0.043	Chloroplast
*ItbMDH7*	OR359914	2536	1065	354	37.28	8.4	29.49	0.177	Mitochondrion
*ItbMDH8*	OR359917	3552	1074	357	37.42	8.36	32.55	0.149	Chloroplast
*ItbMDH9*	OR359924	3580	1074	357	37.42	8.36	32.55	0.149	Chloroplast
*ItbMDH10*	OR359918	2756	999	332	35.54	6.11	31.37	0.045	Cytoplasm

**Table 2:** Ka/Ks analysis and predicted divergence times in three tested species.

Seq_1	Seq_2	Ka	Ks	Ka/Ks	T (Mya)	Duplication Type	Type of Selection
*IbMDH5*	*IbMDH7*	0.12	0.91	0.13	57.2	Segmental	Purify selection
*IbMDH6*	*IbMDH8*	0.11	0.74	0.14	46.4	Segmental	Purify selection
*ItfMDH1*	*ItfMDH3*	0.05	0.81	0.06	50.3	Segmental	Purify selection
*ItbMDH1*	*ItbMDH2*	0.04	0.78	0.06	48.9	Segmental	Purify selection
*ItbMDH3*	*ItbMDH6*	0.09	0.73	0.13	45.4	Segmental	Purify selection

## Data Availability

The data and materials that support the findings of this study are available from the corresponding authors upon reasonable request.

## Supplementary Materials

The following supporting information can be downloaded at: https://zenodo.org/record/8375626 (accessed on 27 September 2023).
